# Comparing 600 years of extremely hot Central European summers to future projections

**DOI:** 10.1038/s41598-026-45507-z

**Published:** 2026-04-01

**Authors:** Laura Lipfert, Ralf Hand, Stefan Brönnimann

**Affiliations:** 1 Institute of Geography and Oeschger Centre for Climate Change Research, University of Bern, Bern, Switzerland; 2 Present Address: Deutscher Wetterdienst DWD, Offenbach, Germany

**Keywords:** Summer heat, Large ensemble, Reanalyes, Palaeoclimate, Climate sciences, Environmental sciences

## Abstract

We use a unique combination of (paleo-)reanalysis (ModE-RA, ERA5) and model simulations (ModE-Sim) to examine extreme summer heat events in Central Europe over the past 600 years (1421-2008) and compare their occurrences to CMIP6 future climate projections. Using a common, fixed climatology, 2003 was identified as the hottest summer of the past 600 years. However, using a moving climatology approach, we identify 1540 (April–September, +2.2 $^\circ$C) and 1590 (June–August, +2.8 $^\circ$C) as the most extreme summer anomalies. The two summers differ in their temporal development, their impacts, and partly their atmospheric circulation patterns. We then analyze similarly anomalous summers in two sets of CMIP6 projections and the ModE-Sim ensemble and connect their occurrence to sea surface temperature anomalies over the North Atlantic. ModE-Sim, which comprises 11,760 model years, produces 0.14% (April-September) and 0.24% (June-August) Central European summers with temperature anomalies from a moving climatology surpassing 1540 and 1590. In CMIP6 projections such anomalies are more frequent, but none reaches the magnitude of the largest June-August anomaly in ModE-Sim which exceeds 4 $^\circ$ $^\circ$C. A similarly extreme anomaly in a future climate could dramatically affect ecosystems and societies. Overall, the combination of reanalysis and model simulations provides a unique and comprehensive framework for understanding the drivers of past and future summer heat extremes.

## Introduction

In the last few decades, Central Europe has been affected by several severe summer heat events with record-breaking climate anomalies^1,2^ associated with massive social, economic and environmental impacts^3,4^. Prominent examples for Central Europe are 2003^5^, 2013^6^, 2015^7^ and the extreme summer of 2018^8^. Research about the extreme Central European summer 2003 revealed that the probability of such an extreme climate anomaly was extremely unlikely even when accounting for climate change and that it can only be explained with increased climate variability under global warming^5^. Consequently, with ongoing global warming and further increasing temperature variability, hot summers over Europe are projected to become more frequent and more extreme^9–11^. However, to test statements about the rarity of current extremes, it is relevant to analyse extremely hot summers in past centuries.

Because extremely hot summers are rare by definition, large samples of summers are required for their study. These can be acquired from long records of past climates^12^ or from model simulations^13,14^. Long observational records or reconstructions^15^ of past climate capture real extremes that had real impacts, but such data are normally not available in the same comprehensive form as climate model data. Model simulations can be used to get insights into the dynamics of such extremely rarely observed events and to understand their driving mechanisms^16^. Furthermore, projections of future climate conditions provide estimates of how frequent and intense extreme summer events may become under continued global warming^1^. Here we use a novel, 600-yr palaeo-reanalysis, a large ensemble of atmospheric model simulations of the past 600 years as well as simulations of future climates to answer the following research question: How unusual are the hot summers of the 20^th^ and 21^st^ century in light of extreme summer in the past 600 years, and how do they compare with simulated future summers?

A well-known heat summer in Central Europe occurred in 1540 during a severe, 11-month long drought event^17,18^. The heat and drought in 1540 were longer-lasting than the hot summer of 2003, affecting a larger area^17^ and in some areas of Central Europe (i.e. Switzerland) possibly even warmer than 2003^19^. Several regions experienced increased mortality due to famine, illness and water scarcity and the heat and precipitation deficit caused widespread forest fires across Europe^20^. Furthermore, the preceding decade (1531-1540) was extraordinary warm and dry, which further amplified the societal stress.

Studying such rare past events can be beneficial to learn more about potential future extremes, but the lack of observational data poses a limit to the understanding of this connection. Previous studies have investigated past extreme summers in Europe mainly using data from single stations^6^ or annually resolved proxy-based reconstructions^15,21^, but systematic, multi-variable studies on monthly resolution, which are necessary to describe atmospheric processes, are lacking.

Previous research shows that hot Central European summers are related to a trough–ridge pattern in the North Atlantic–European sector and a poleward shifted jet stream, with elements of the Summer North Atlantic Oscillation^22^ and the Summer East Atlantic Pattern^23^, which can be modified by North Atlantic sea surface temperatures (SSTs)^12^. While multiple previous Central European heat events were connected to negative SST anomalies in the North Atlantic^12^, model-based assessments also show a connection a positive state of the Atlantic Multidecadal Variation and extreme summers over the Euro-Mediterranean region^13,24^. In this study we analyse past extremely hot summers in the light of atmospheric circulation patterns and SSTs.

For this, we take advantage of the new ModE (Modern Era) dataset family^25^, which provides monthly, global fields of many variables of the past 600 years (1421–2008). The main product, ModE-RA, is a 20-member paleo-reanalysis that combines information from model simulations and observations with an off-line data assimilation approach. It is constrained by perhaps the most comprehensive collection of observations available and thus captures historical extremes as realistically as possible. We also use a 20 member set of the underlying model simulations ModE-Sim. These are atmosphere-only simulations with prescribed SSTs (from an ensemble of reconstructions) that vary across ensemble members^26^. Furthermore, ModE-Sim uses prescribed volcanic forcing, solar irradiance, greenhouse gas concentrations, tropospheric aerosols and land surface conditions that are identical for all members^26^. While the model simulations lacks coupling with ocean dynamics, it provides a large sample size (11,760 model years). In a previous study, we have shown that the ModE-Sim ensemble can be used to analyze extremely hot summers in the more recent past (1850–2008)^14^. Finally, we use ModE-RAclim, which assimilates the same observations into a random sample of 100 priors, drawn from all members and model years of ModE-Sim. This 100-member reanalysis thus does not see the time-varying forcings of the atmospheric model (SSTs, volcanic aerosols etc.) and hence comparing ModE-RA, ModE-RAclim and ModE-Sim allows disentangling the contributions of observations and forings to the reanalysis. A more detailed description of the used datasets can be found in the data section and an overview in Table 1.

**Table 1:** Overview of the datasets used in this study.

	ModE-RA	ModE-RAclim	ModE-Sim
members	20	100	20 (used here)
time dependent forcing	yes	no	yes
assimilation	yes	yes	no

In addition, we use the ERA5 reanalysis and CMIP6 simulations. The focus is on temperature averaged over Central Europe (0–20$^\circ$ E,47–57$^\circ$ N). In addition to a fixed climatology (1950–2008, which is the longest joint period), we use a moving climatology approach relative to a LOESS (locally estimated scatterplot smoothing) regression that approximately corresponds to a 30-yr average to calculate the anomalies. Then we identify the most extreme summers in the past 600 years as well as in CMIP6 projections. In addition to temperature, we address precipitation, atmospheric circulation, and SST during these events.

## Results

### Past Central European summers in ModE-RA and ModE-Sim

**Fig. 1:**
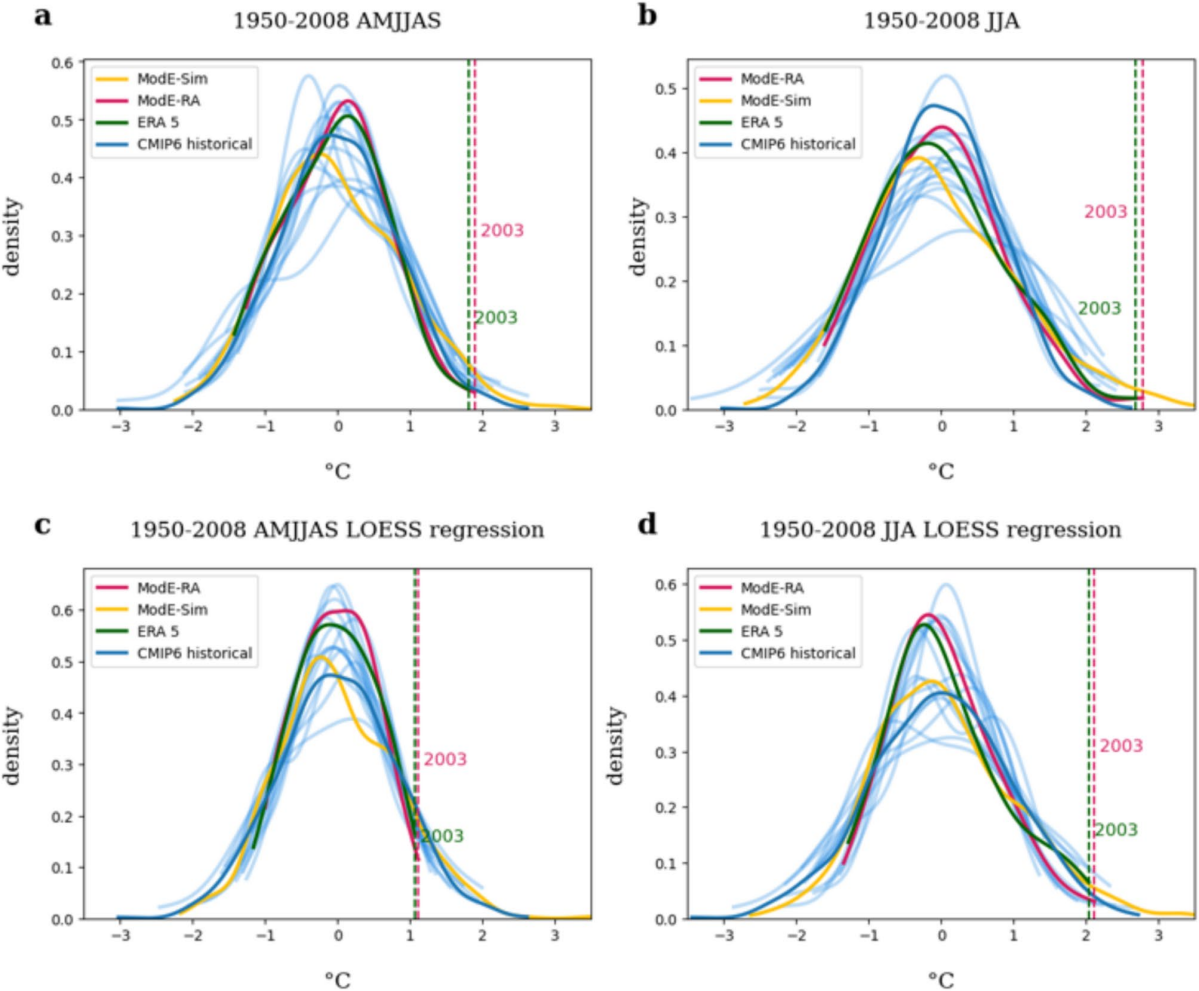
1950–2008 distributions of Central European (0–20$^\circ$E,47–57$^\circ$N) 2 m temperature anomalies for ModE-Sim (yellow), ModE-RA (pink), ERA5 (green), CMIP6 historical simulations (blue shaded lines) and CMIP6 multimodel mean (blue) for (**a**) April–September and (**b**) June–August with fixed climatology (1950–2008) and (**c**) April–September and (**d**) June–August relative to a moving climatology. Vertical lines indicate the strongest anomaly in ModE-RA (pink) and ERA5 (green).

To evaluate the datasets, we first compare the distribution of the ModE-RA and ModE-Sim summer temperature anomalies to ERA5 and the different CMIP6 historical simulations for the overlapping time-period 1950-2008 (Fig. 1). In addition to the standard summer season, June to August (JJA), we also investigate extreme temperature anomalies over an extended summer season, April to September (AMJJAS). The extended summer season corresponds to the growing season; events affecting the entire growing season might be particularly relevant for climate impacts.

The strongest anomaly for both AMJJAS and JJA in ModE-RA and ERA5 occurs in 2003, regardless of whether a fixed or moving climatology is used. In all cases, the summer of 2003 appears slightly more extreme in ModE-RA than in ERA5 (Fig. 1a,b). For the fixed 1950–2008 climatology, the JJA anomaly in ModE-RA reaches 2.78 $^\circ$C, which is consistent with previous studies of the 2003 heatwave, although the strongest anomalies occurred south-west of our study region^5,27^. The CMIP6 historical simulations and ModE-Sim, which are not constrained by atmospheric observations, exhibit a somewhat wider range of temperature anomalies, including events that exceed the 2003 anomaly in both seasons. Overall, the agreement between ModE-RA, ModE-Sim, the CMIP6 multi-model ensemble, and ERA5 is good, and it further improves when using a moving climatology (Fig. 1c,d).

The comparison of hot extremes using qq-plots shows a generally consistent upper-tail behavior between ERA5 and ModE-RA, but larger deviations for ModE-Sim and the CMIP6 ensemble (Supporting Information Fig. S1). These discrepancies are expected, as ModE-Sim and CMIP6 are not constrained by atmospheric observations and therefore display a wider and less tightly aligned distribution of extreme values. Despite these differences, the overall level of agreement remains sufficient for our purposes. We therefore continue to use ModE-RA and ModE-Sim to analyze extreme Central European summers over the past 600 years and compare their occurrence with future projections. In the results section, we focus on anomalies relative to a moving climatology.

**Fig. 2:**
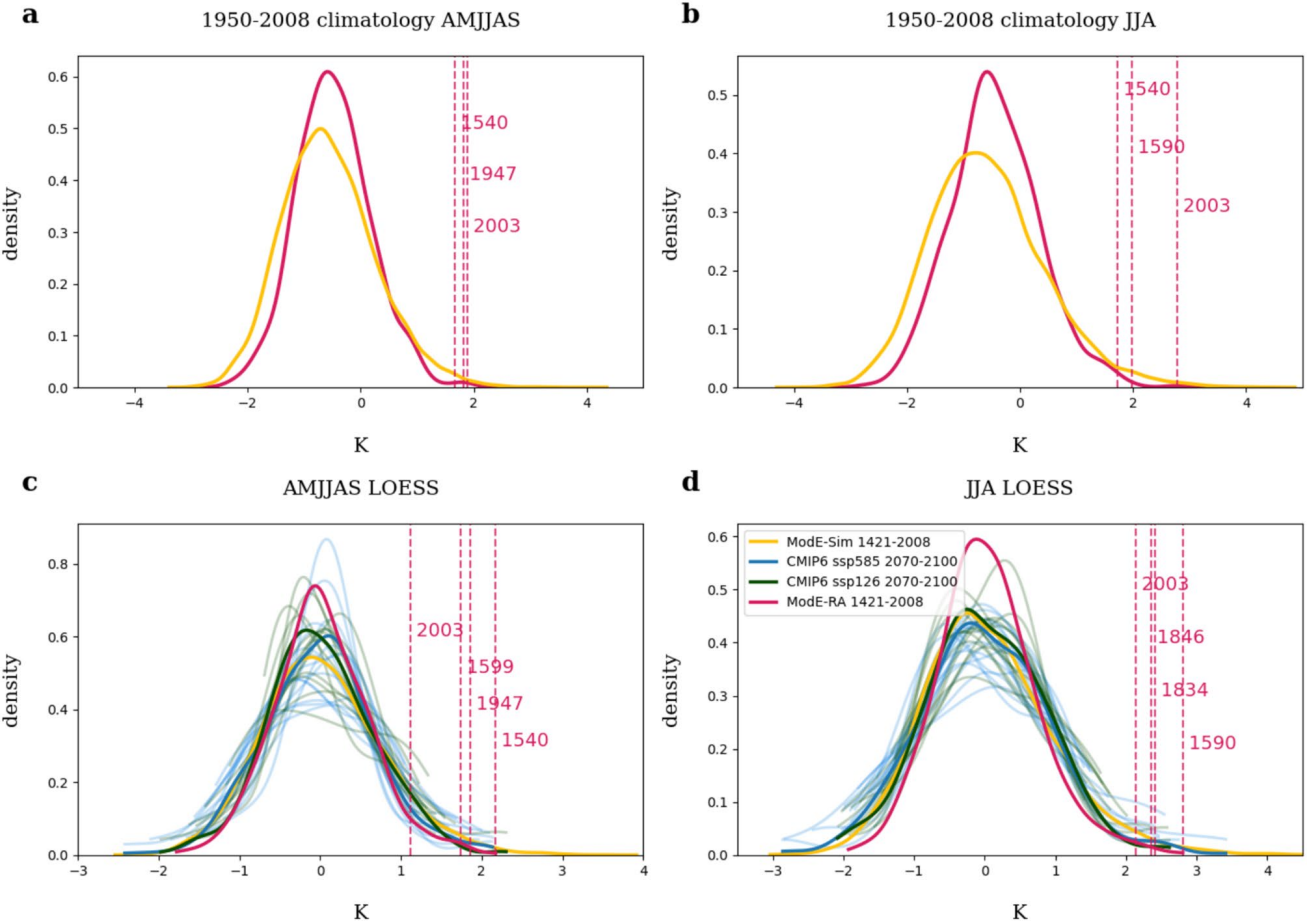
Distributions of Central European (0–20$^\circ$E,47–57$^\circ$N) 2 m temperature anomalies for 1421–2008 ModE-Sim (yellow), 1421–2008 ModE-RA (pink) for (**a**) April–September and (**b**) June–August relative to a fixed climatology and (**c**) April–September and (**d**) June–August temperature anomalies relative to a moving climatology. Pink vertical lines indicate the strongest three anomalies in ModE-RA and in addition the summer of 2003. Legend for all subplots in (**d**).

When investigating Central European summers during the full ModE-RA period 1421-2008 (Fig. 2a,b), the warmest summer for both AMJJAS and JJA is no longer 2003, although it is still above the 99th percentile (5th hottest) for JJA and slightly above the 90th percentile (25th hottest) for the extended summer season, AMJJAS. Rather, the hottest extended summer season is 1540, followed by 1947 and 1599. For JJA, the hottest summer is 1590, followed by 1834 and 1846 (Fig. 2c,d). For 1540, the AMJJAS anomaly over Central Europe averages 2.17 $^\circ$C (compared to only 1.12 $^\circ$C in 2003) and for 1590, the average temperature JJA anomaly is 2.80 $^\circ$C (2.13 $^\circ$C in 2003). This shows that ModE-RA is capable of reproducing past extreme events over Central Europe that were (in the context of their surrounding climate) even more extreme than the summer of 2003. While the 1540 event has been extensively studied, the 1590 summer is poorly known. The two extreme summers will be discussed further in the following section. The summer of 1947 and its impacts were addressed previously^28^.

To compare temperature anomalies across different centuries, we use a LOESS regression. It has has been proposed as an effective method to estimate non linear trends and especially to extend a trend line towards the beginning and the end of a time series (in comparison to running averages)^29^. However, due to the local fitting approach there is some sensitivity of this method to outliers. To address this, we conducted a sensitivity test by recalculating the LOESS regression with and without the most extreme years 1540 and 1590. The results showed minimal differences, with the largest temperature change being only 0.02 $^\circ$C (range: 0.004–0.02 $^\circ$C). This demonstrates that the LOESS regression used in this study is a suitable tool to analyze extreme summers of the past 600 years and more robust to outliers than other traditional regression methods.

### The hot summers of 1540 and 1590

**Fig. 3:**
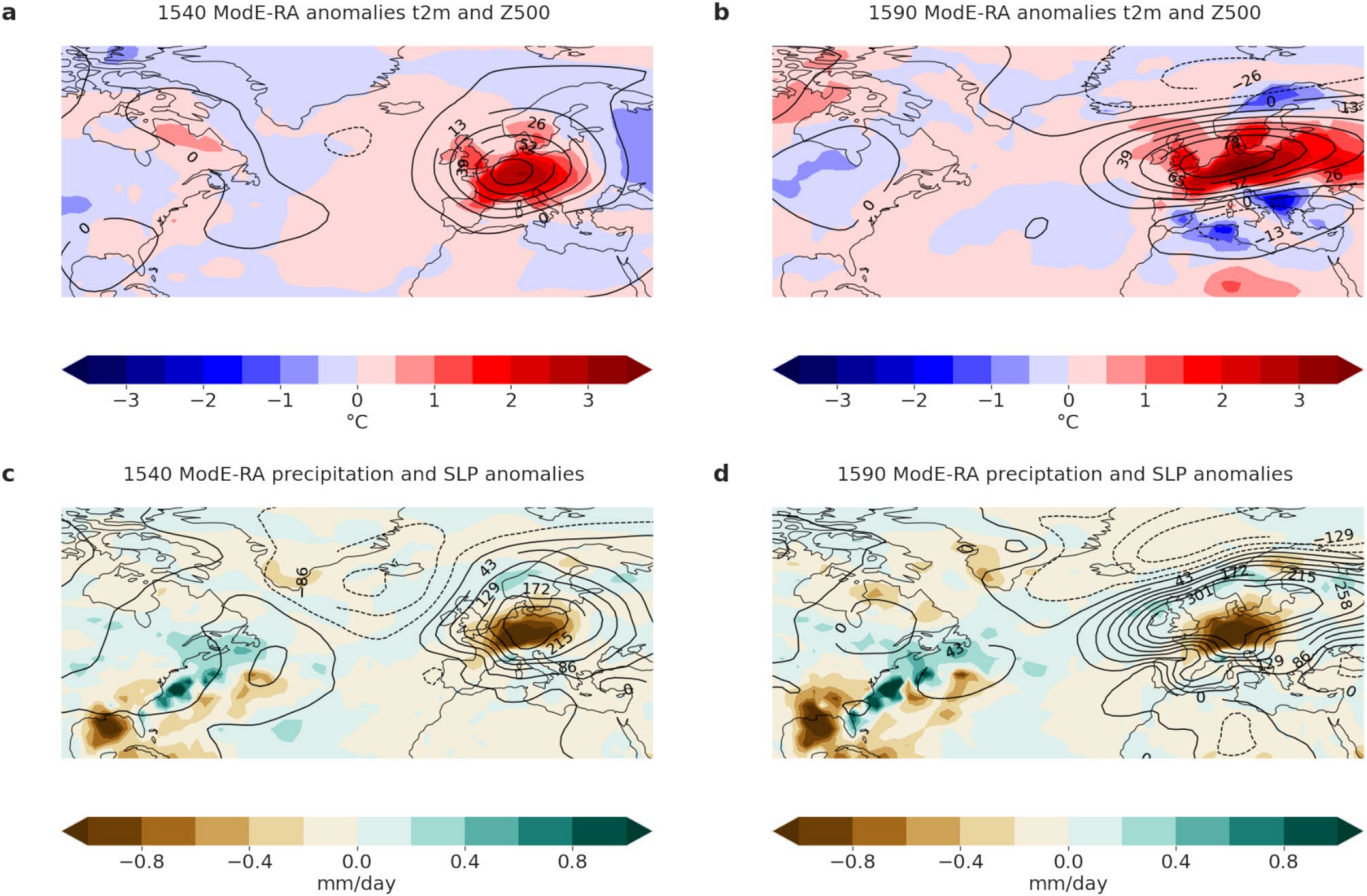
(**a**) ModE-RA 2 m temperature (shadings) and 500 hPa geopotential height anomaly (contours with intervals of 13 gpm, negative anomalies dashed) for April-September 1540 (**b**) ModE-RA 2 m temperature and 500 hPa geopotential height anomaly for June–August 1590 (**c**) ModE-RA total precipitation (shadings) and sea level pressure anomaly (contours with intervals of 43 Pa, negative anomalies dashed) for April–September 1540 (**d**) ModE-RA total precipitation and sea level pressure anomaly for June–August 1590.

The year 1540 is known for its extraordinarily hot summer over Central Europe^30^, which marked the culmination of a severe 11-month long drought event^17,18^. However, not much is known about the underlying atmospheric dynamics. We therefore investigate the spatial distribution of the anomalies in temperature, 500 hPa geopotential height (Z500), total precipitation and sea level pressure (SLP) in more detail (Fig. 3a,c).

In ModE-RA the positive AMJJAS 2 m temperatures anomaly (calculated with the same moving climatology) extends from Italy up to Southern Scandinavia and from the Atlantic Ocean up to Western Ukraine reaching up to 3 $^\circ$C (Fig. 3a) and is accompanied by a positive Z500 anomaly. An index of the latitude of the Atlantic-European jet at 500 hPa in ModE-RA^31^ shows that the jet was persistently and strongly, but not extremely, displaced northward for an entire year (Nov. 1539 to Oct 1540), in line with the reports of an 11-month drought. When comparing the temperature anomalies in ModE-RA, ModE-Sim and ModE-RAclim, it becomes clear that there is no influence from the ModE-Sim SST forcings on the 1540 heat event (Supporting information Fig. S2). This absence suggests that it was likely not forced by the model boundary conditions but rather driven by internal atmospheric variability. Precipitation in AMJJAS shows a negative anomaly of up to 1 mm/day mainly over Central Europe, while there is a strong positive SLP anomaly reaching from Italy up to Scandinavia including the British Isles. A negative SLP anomaly can be found over Iceland and the North Atlantic (Fig. 3c). This SLP pattern is comparable to the pattern for the summer of 1540 found in other studies^18^.

While 1540 is known as an extraordinary hot and dry summer in an overall very dry decade (1531–1540)^18^, there is almost no research about the hot summer of 1590 which is the largest JJA anomaly in ModE-RA relative to a moving climatology (the second largest in absolute temperature). Investigating documentary data from thw Czech Republic there is evidence that there was a severe drought in 1590 as well as extreme heat that caused multiple forest fires^32^. However, contrary to 1540, June-August 1590 was an outlier in an overall wetter and colder period. Consequently there were fewer historical records of severe impacts which might also explain the scarcity of literature about this event. The ModE-RA gridded reanalysis dataset now allows a more detailed analysis. The JJA 2 m temperature anomaly (relative to a moving climatology) extends more to the East and less to the South than in 1540, reaching temperature anomalies of up to 3 $^\circ$C in large parts of Central Europe (Fig. 3b). The Z500 anomaly also extends all the way into Eastern Europe and Russia. The jet latitude index^31^ reached even much higher values than in 1540, but only in June and July. The summer was also very dry, but for a shorter period than in 1540 (anomalies are of similar magnitude). There were 28 days with precipitation (10 in June, 5 in July and 13 in August) in Fürstenfeldbruck near Munich, which is slightly more than half the number of days in the other six years of this seven-year record^33^. Comparing the temperature anomalies in ModE-RA, ModE-Sim and ModE-RAclim reveals that also for the summer of 1590, there is no influence from the ModE-Sim SST forcings (Supporting Information S2). The positive SLP anomaly also extends further east, however, the negative total precipitation anomalies are most pronounced over Central Europe, similar to 1540 (Fig. 3d).

Previous research on extreme summer temperatures in Central Europe shows that heat extremes occur together with strong Z500 anomalies^16^. Unsurprisingly, ModE-RA shows that this is also true for the extreme seasons 1540 and 1590. For 1540, the strongest temperature anomaly is found in the center of the Z00 anomaly. While blocking cannot directly be assessed in a seasonal ensemble mean, some individual members show an Omega blocking pattern for the extended summer season of 1540. For 1590, the highest temperature anomaly is a bit southeast of the highest Z500 anomaly. The ensemble mean Z500 anomaly hints at a Rex/Dipole blocking pattern, which is again supported by investigating the individual members. Such circulation patterns, including Omega and Rex/dipole blocking configurations, are well-known drivers of extreme summer heat over Europe^16,31^. Overall, Fig. 3 demonstrates the ability of ModE-RA and ModE-Sim to reproduce the expected correlation between positive Z500 anomalies and extreme heat events over the past 600 years, increasing the reliability of the datasets to analyze historical and projected heat extremes.

### Extreme European summers and heatwaves in ModE-Sim

**Fig. 4:**
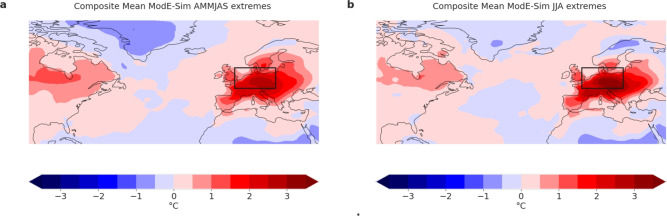
Composite Means of all ModE-Sim years (all 20 members) with (**a**) 2m temperature April–September anomalies greater than ModE-RAs 1540 anomaly (**b**) 2m temperature June–August anomalies greater than ModE-RAs 1590 anomaly.

The 11,760 model years of ModE-Sim feature even more extreme summers than those of 1540 and 1590 (Fig. 2c,d), which here are analysed spatially (Fig. 4). Searching for Central European summer temperature anomalies relative to a moving climatology that are equal to or higher than the anomalies for 1540 or 1590 (2.17 or 2.80$^\circ$C), we find 17 and 29 summers, corresponding to 0.14% and 0.24% of all summers, respectively. The largest JJA temperature anomaly even exceeds 4 $^\circ$C. The distributions (Fig. 2) show that the ModE-Sim ensemble can simulate extreme summer temperature anomalies that are on the far-right edge of the CMIP6 simulations. A Levene’s test shows that the standard deviations of ModE-Sim (Table 2) are significantly higher than those of the CMIP6 historical simulations for AMMJAS, while for JJA the difference is not statistically significant. Note that ModE-Sim also has a statistically significantly higher standard deviation than ModE-RA for both seasons.

**Table 2:** Standard deviations $\sigma$ for Central European summer temperatures anomalies relative to a moving climatology (distributions shown in Fig. 2).

	April-September	June-August
$\sigma$ ModE-RA	0.59	0.70
$\sigma$ ModE-Sim	0.76	0.94
$\sigma$ CMIP6 ssp126	0.64 (0.51–0.81)	0.83 (0.63–0.99)
$\sigma$ CMIP6 ssp585	0.69 (0.54–0.84)	0.90 (0.75–1.25)

For the different CMIP6 scenario runs the first value is the median of all models. In addition, the highest and lowest values are shown in brackets.

The composite means of the selected summers reveal that positive temperature anomalies are largest over continental Europe and do not extend far into the North Atlantic or the Mediterranean Sea (Fig. 4a,b). There are no strong connections to SST anomalies over the North Atlantic that would be visible in the composite mean, but analyzing the individual cases, we find both strong positive and strong negative SST anomalies in the North Atlantic that support a connection to hot summers over Central Europe (Supporting information Figs. S3, S4). In another study, analyzing the relationship between heatwave days and SSTs using ModE-Sim, we also found a connection between positive anomalies in the North Atlantic and more heatwave days over the Euro-Mediterranean region^14^.

### Future extreme European summers in the CMIP6 multimodel ensemble

To compare the historical extreme summer seasons (ModE-RA and ModE-Sim) to potential future extremes, we analyze the distribution of the CMIP6 multimodel ensemble for the two warming scenarios ssp585 (additional radiative forcing of 8.5 W/m$^2$ by the year 2100, high emission scenario) and ssp126 (additional radiative forcing of 2.6 W/m²$^2$ by the year 2100, scenario with strong climate protection). Note that we selected the CMIP6 simulations used in this analysis according to their ability to simulate European summer temperatures^34^. For CMIP6, we plot the distributions for the years 2070-2100 relative to a moving climatology. While there are large differences between the scenarios in the absolute summer temperatures (not shown), these are relatively small when using a moving climatology approach. Standard deviations are higher for ssp585 than for ssp126, both are slightly higher than ModE-RA and slightly lower than ModE-Sim (Table 1). However, it is important to keep in mind that ModE-Sim atmospheric simulations are constrained by SSTs while the CMIP6 models are fully coupled.

**Fig. 5:**
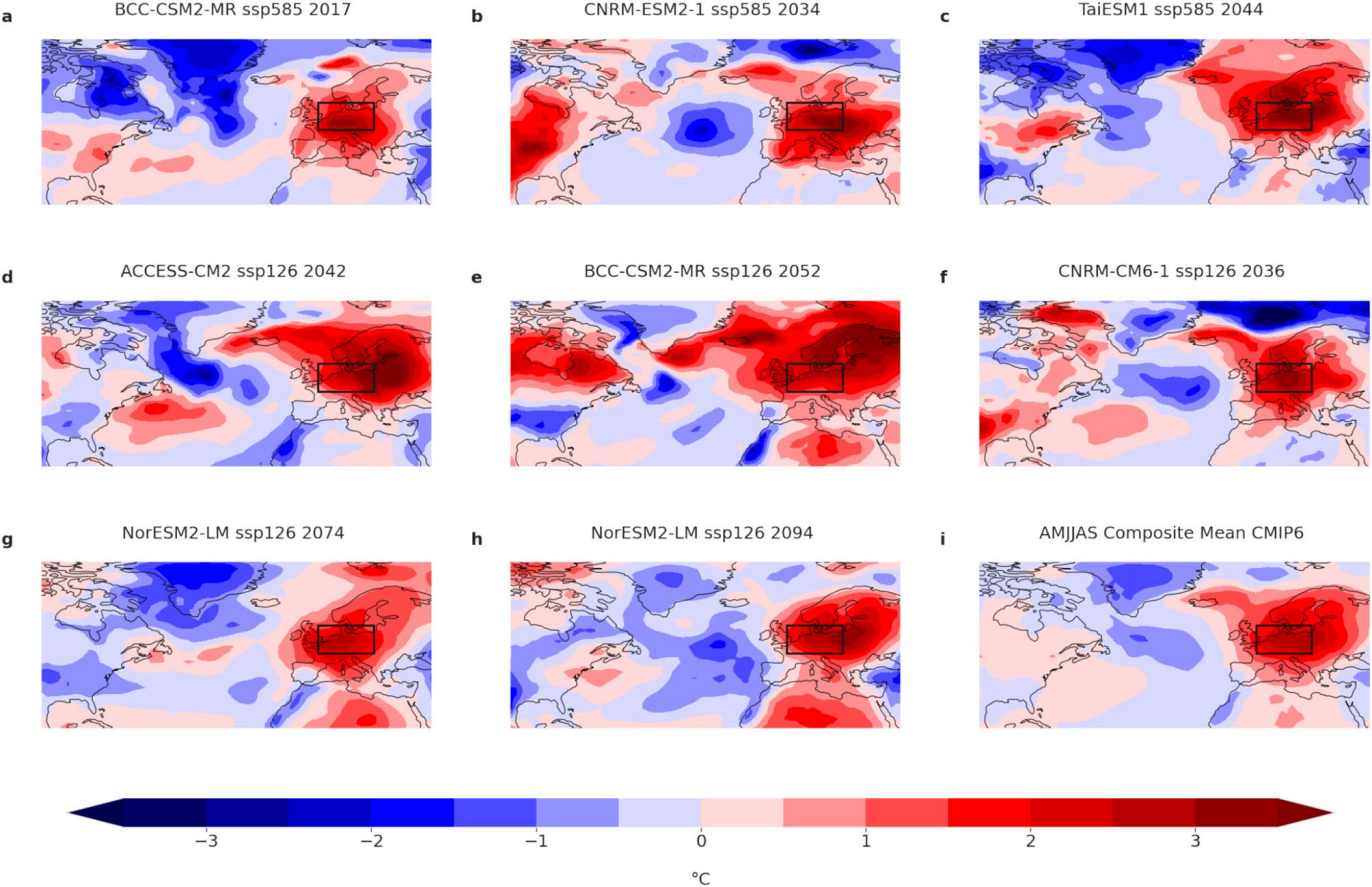
(**a**–**h**) 2 m temperature fields for all CMIP6 simulations with anomalies over Central Europe(0–20$^\circ$E,47–57$^\circ$N) greater than ModE-RAs April–September 1540 anomaly (2.17K). (**i**) Composite mean of all CMIP6 simulations with anomalies over Central Europe greater than ModE-RAs 1540 anomaly.

Based on this agreement, we can now compare past and future anomalies. The anomaly of the hottest extended summer season in ModE-RA, 1540, is above the multi-model mean of the 99^th^ percentile for both scenarios, ssp585 and ssp126. The same is true for the strongest JJA anomaly in ModE-RA, 1590 (Fig. 2c,d). Few stronger anomales are found in the CMIP6 multi-model ensemble. For AMJJAS, in all 29 analyzed CMIP6 simulations (both scenarios), we find eight summers where the anomaly over Central Europe relative to a moving climatology was larger than the ModE-RA anomaly in 1540 (2.17 $^\circ$C) (Fig. 5), corresponding to 0.9%. In all cases, the positive anomalies extend over a large region of Europe and for some cases far into the North Atlantic Ocean up to Iceland (Fig. 5b–e). A pattern that appears to be related to hot summers over Europe is a negative SST anomaly over the North Atlantic south of Greenland. This is also visible in the composite mean of all CMIP6 extreme summers (Fig. 5i). Negative SST anomalies in the North Atlantic have been linked to several Central European heat events^12^ and our analysis contributes to this assessment. For June-August extreme summers in the CMIP6 multimodel ensemble (warmer or equal than 1590, 2.80 $^\circ$C: seven summers or 0.8%), the negative SST anomalies in the North Atlantic are a bit less pronounced but also visible in the composite mean (Supporting information Fig. S5).

Although rare, anomalies of the same magnitude as in 1540 and 1590 do occur in CMIP6 future simulations. Given the several-degree increase in the underlying moving climatology, such summers will be far hotter in absolute temperatures than 1540 or 1590 (Fig. 2a,b), which can have devastating consequences for ecosystems and societies.

## Discussion and conclusion

We show that a 600-year long monthly paleo-reanalysis ModE-RA and the underlying atmospheric model simulations ModE-Sim realistically simulate Central European heat extremes. Using a moving climatology calculated with LOESS regressions, the hottest summers identified are 1540 and 1590, even surpassing the well-known 2003 hot summer when considering their surrounding climatic context. Both years featured severe heat anomalies driven by a poleward-shifted Atlantic-European jet and blocking high pressure systems over Central Europe and were accompanied by a strong precipitation deficit, but their temporal development differs markedly: 1540 was a prolonged drought event peaking in a heat summer and it followed an extremely dry decade, whereas 1590 was a short but extremely hot summer embedded in a rather cool and wet decade. Although 1540 was identified before as European mega-drought with regionally higher temperature anomalies than 2003^19^, there are quite some uncertainties attached to the reconstructed temperature anomalies as tree-rings or grape harvest dates may fail to detect such record-breaking climatic outliers^20^. ModE-RA combines all available documentary and proxy-based evidence with model simulations and SST reconstructions to simulate 16^th^ century temperatures and also atmospheric circulation. Note, however, that during the 16^th^ century, there were no observations over the ocean and the SST reconstructions for this period are based on an analogue approach^35^. The absence of a signal for 1540 in the ModE-Sim ensemble mean (Supporting Information Fig. S2) could therefore reflect not only the dominance of internal atmospheric variability, but also uncertainties in the prescribed SST boundary conditions. Investigating the precipitation patterns (Fig. 3), it appears that the same SST analogue was chosen for the years 1540 and 1590, resulting in similar precipitation patterns in places where no observations are available. While this hints at a limitation of the SST reconstruction method, it does not influence the reliability of the datasets for capturing key circulation patterns and temperature signals, particularly in Europe, where many observations enter the reanalysis. We can consequently still provide a comprehensive assessment of past climate extremes and contribute to a better understanding of rare climate extremes like 1540 or even 1590, which have been been sparingly covered in previous research^32^.

As to a possible increase in temperature variability, we find limited evidence for an increase in the most extreme anomalies. Magnitudes comparable to 1540 and 1590 are very rare also in a future climate. The strongest event in 588 years of ModE-RA corresponds to one event per 692 or 406 years of ModE-Sim for AMJJAS and JJA and one per 112 or 128 years of CMIP6, with no difference between ssp585 and ssp126. This makes the events of 1540 and 1590 still relevant study cases for today. The 11,760 model years of ModE-Sim produce even more extreme summer temperature anomalies than those projected by the CMIP6 multimodel ensemble, the largest exceeding 4 $^\circ$C. Combining and comparing the model simulations with techniques like ensemble boosting^36^ could create a unique opportunity to study future extreme events. However, there is also a possibility that ModE-Sim may overestimate these extremely warm summers, highlighting the need for careful interpretation of results when using this model ensemble. It is also important to note that the CMIP6 single scenario runs might not be able to reproduce as many extreme events as a 20-member, 600-year ensemble. Furthermore, ModE-Sim is in contrast to the CMIP6 models not a coupled earth system model but rather an atmosphere-only model with prescribed SST, and land-surface conditions^26^. The heat capacity of the ocean is infinite in ModE-Sim as there is no SST damping and no ocean/atmosphere coupling. This could potentially lead to more extreme summers and should be kept in mind when interpreting the results. While SSTs and volcanic aerosols vary across ensemble members in ModE-Sim, the anthropogenic forcings are held constant, allowing the detrended ensemble mean to primarily reflect the influence of SST and volcanic variability. However, this does not strictly rule out the possibility that post-1850 anthropogenic forcings could co-vary with SST and contribute to the identified signals.

For extreme summers over Central Europe similar to 1540 and 1590 in the CMIP6 multimodel ensemble, we find a connection to a negative SST anomaly over the North Atlantic. Especially for Central Europe, it is known that negative SST anomalies in the North Atlantic are often followed by positive summer temperature anomalies^12^. However, previous research also shows that there is a connection between a positive state of the Atlantic Multidecadal Variation, resulting in a warmer than average North Atlantic, and hot summers over the whole Euro-Mediterranean region^13,24^. This is also supported by our previous research on heatwaves in ModE-Sim where we find a connection between positive North Atlantic SST anomalies and increased heatwave days for the whole Euro-Mediterranean region^14^. In this study, the ModE-Sim composites for extreme summer season do not show a strong SST anomaly over the North Atlantic. However, for the individual extreme summers we see both: strong negative but also strong positive anomalies over the North Atlantic that could potentially be connected to Central European hot summers. Furthermore, the summer of 1590 and particularly that of 1540 were preceded by dry springs, the effect of which is a topic to be addressed in future studies.

In summary, this study uniquely combines a paleo-reanalysis (ModE-RA), a reanalysis (ERA5) and model simulations (ModE-Sim, CMIP6) to analyze extreme summer heat events in Central Europe over the past 600 years using a moving climatology approach. We find very extreme summers in the observational period that were far more pronounced than that of 2003 and are comparable in anomalies to the hottest summers in climate scenario simulations. Moreover, the two summers studied, 1540 and 1590, highlight different temporal developments, to which different impacts are related, whereas the dynamical situation was similar though with some differences. Together, this contributes a deeper understanding of such extreme summers and shows that ModE-Sim and ModE-RA are valuable for exploring extreme climatic anomalies and their drivers.

## Datasets and methods

### Datasets

ModE-RA (Modern Era Reanalysis) is a 20-member gridded global monthly paleo-reanalysis that covers the period 1421-2008 with a horizontal resolution of approximately 1.8$^\circ$ by 1.8$^\circ$ (T63)^25^. Monthly and seasonal climate information from tens of thousands of series including natural proxies, documentary data, and instrumental data are assimilated off-line into an ensemble of transient model simulations ModE-Sim (see next paragraph) using an Ensemble Kalman Filter approach. The background error covariance matrix is calculated from the ensemble but blended with a covariance matrix calculated from a random sample of 100 years from the ModE-Sim in order to make the covariance structure smoother. The same assimilation approach is used to generate ModE-RAclim, except that this version uses a random sample of 100 years from the ModE-Sim ensemble as prior and also for the background error covariance matrix. As both the prior and the background error covariance matrix are time-invariant, ModE-RAclim does not see the time-dependent forcing. In contrast, ModE-RA utilizes 20 distinct transient members of ModE-Sim, preserving the effects of external forcings within the model simulations^25^.

In addition to the reanalysis, we analyze ModE-Sim (Modern Era Simulations). We use sets 1420-3 and 1850-1, i.e., the 20-member ensemble that underlies ModE-RA (note that there are additional sets). ModE-Sim is based on the atmospheric general circulation model ECHAM6^26^. As ocean boundary conditions the model uses different realizations of HADISST2^37^ and, to extend the available forcing data, recombinations of them^26^. For the earlier period before 1850 the model ensemble uses SST reconstructions^35^. For the radiative (and volcanic) forcings, the standard PMIP4^38^ input is used. The land-surface conditions for ECHAM6 are provided by the integrated land-surface model JSBACH^39^. In addition, ModE-Sim also includes anthropogenic forcings (greenhouse gases and ozone) that are identical for all ensemble members. All ensemble mean products (ModE-RA, ModE-RAclim, ModE-Sim) can be easily accessed and analysed online at https://mode-ra.unibe.ch/climeapp/. To compare heat extremes in ModE-RA and ModE-Sim to the more recent past and to validate whether our datasets provide a realistic representation of Central European summers, we use the ERA5 daily mean surface temperature from 1950 to 2008 from the ECMWF^40^.

We compare Central European summers in ModE-RA to the future climate projections from the Scenario Model Intercomparison Project (ScenarioMIP) for CMIP6^41^. We decided to compare to two different climate scenarios ssp585, which consists of an additional radiative forcing of 8.5 W/m²$^2$ by the year 2100 (high-emission scenario), and ssp126, consisting of an additional radiative forcing of 2.6 W/m²$^2$ by the year 2100 (scenario with strong climate protection). We selected a total of 16 models from the CMIP6 database based on their availability (both scenarios and historical simulations) and their ability to reproduce realistic Central European summer temperatures^34^. For validation of extreme summers in the past, we further compare ModE-Sim and ModE-RA to the CMIP6 historical simulations.

### Methods

The annual time series of averaged Central European (0–20$^\circ$ E,47–57$^\circ$ N) 2 m air temperature for the two season AMJJAS and JJA were expressed relative to two climatologies: a fixed baseline climatology covering 1950 to 2008 (i.e. the overlap between ERA5, ModE-RA and all CMIP6 historical runs) and a moving climatology. For the moving climatology, we use a LOESS (locally weighted scatterplot smoothing) algorithm^42^. The LOESS method consists of computing a series of local linear regressions, with each local regression restricted to a window of t-values. We choose the smoothing parameter of the regression to achieve approximately 31-year moving windows. The advantage of a LOESS regression is that we can also investigate hot summer anomalies at the beginning and the end of the time series and thereby increase the amount of data that can be used to investigate extreme European summers. Previous research also promotes LOESS as an alternative to the traditional IPCC anomaly calculation methods as it results in lower biases^43^. Note that there are other ways of combining ensemble simulations with observations such as the UNSEEN approach that is used for estimating the chance of an observed extreme meteorological event by combining it with a large, initialised ensemble of simulations^44^.

To investigate extreme summers that are comparable to 1540 (1590), we filtered the ModE-Sim ensemble as well as the CMIP6 multimodel ensemble for all years where the anomalies were larger than the ModE-RA ensemble mean LOESS anomaly 2.17 $^\circ$C (2.80 $^\circ$C) and computed composite means. Although we use near-surface air temperature data for the model simulations and the CMIP6 projections, they are very similar to SSTs and confirm a link between the North Atlantic and hot summers over Central Europe. To justify the use of 2 m air temperature as a proxy for SST, we include a n figure showing the strong correlation between SSTs and near-surface air temperatures over the North Atlantic in ModE-Sim (Supporting Information Fig. 6).

## Supplementary Information

Supplementary Information.

## Data Availability

The ModE-RA datasets as well as the ModE-Sim simulations can be obtained via the World Data Center for Climate (https: //www.wdc-climate.de/ui/project?acronym=ModE). The CMIP6 simuations can be accessed via the DKRZ (https://esgf-metagrid.cloud.dkrz.de/projects/cmip6-dkrz/) and the ERA5 simulations can be downloaded from the Climate Data Store (https://cds.climate.copernicus.eu/datasets/reanalysis-era5-single-levtab=overview).
