# Supplementary material for: Comparing 600 years of extremely hot Central European summers to future projections
Source: Sci Rep. 2026 Apr 1;16:15278. doi: 10.1038/s41598-026-45507-z (PMC13181032; doi:10.1038/s41598-026-45507-z)
Supplement: Supplementary file 1 — Supplementary Information. [file 41598_2026_45507_MOESM1_ESM.pdf]

Comparing 600 years of extremely hot Central European summers to future projections

Laura Lipfert, Ralf Hand, Stefan Brönnimann

February 8, 2026

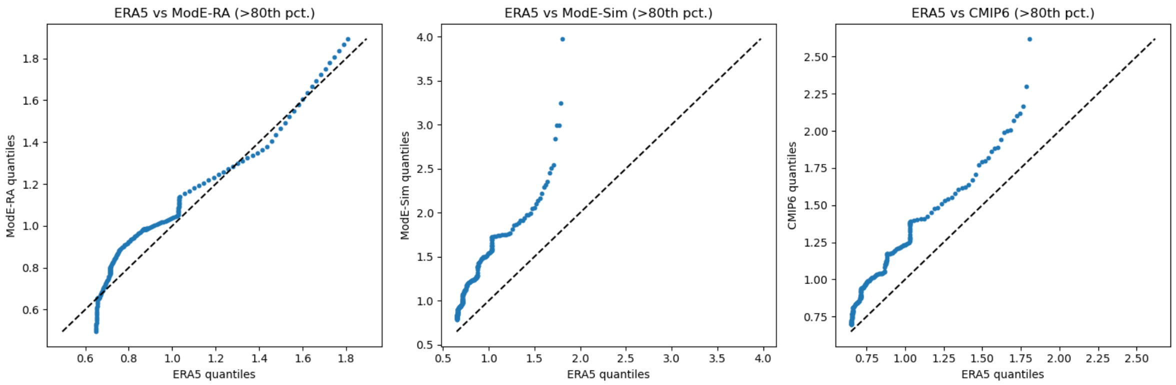

Figure S1: Quantile-quantile plots comparing the upper-tail (above 80th percentile) summer temperature extremes (April-September) of ERA5 with a) ModE-RA, b) ModE-Sim, and c) CMIP6 historical simulations. The dashed line represents the 1:1 relationship, indicating perfect agreement between datasets.

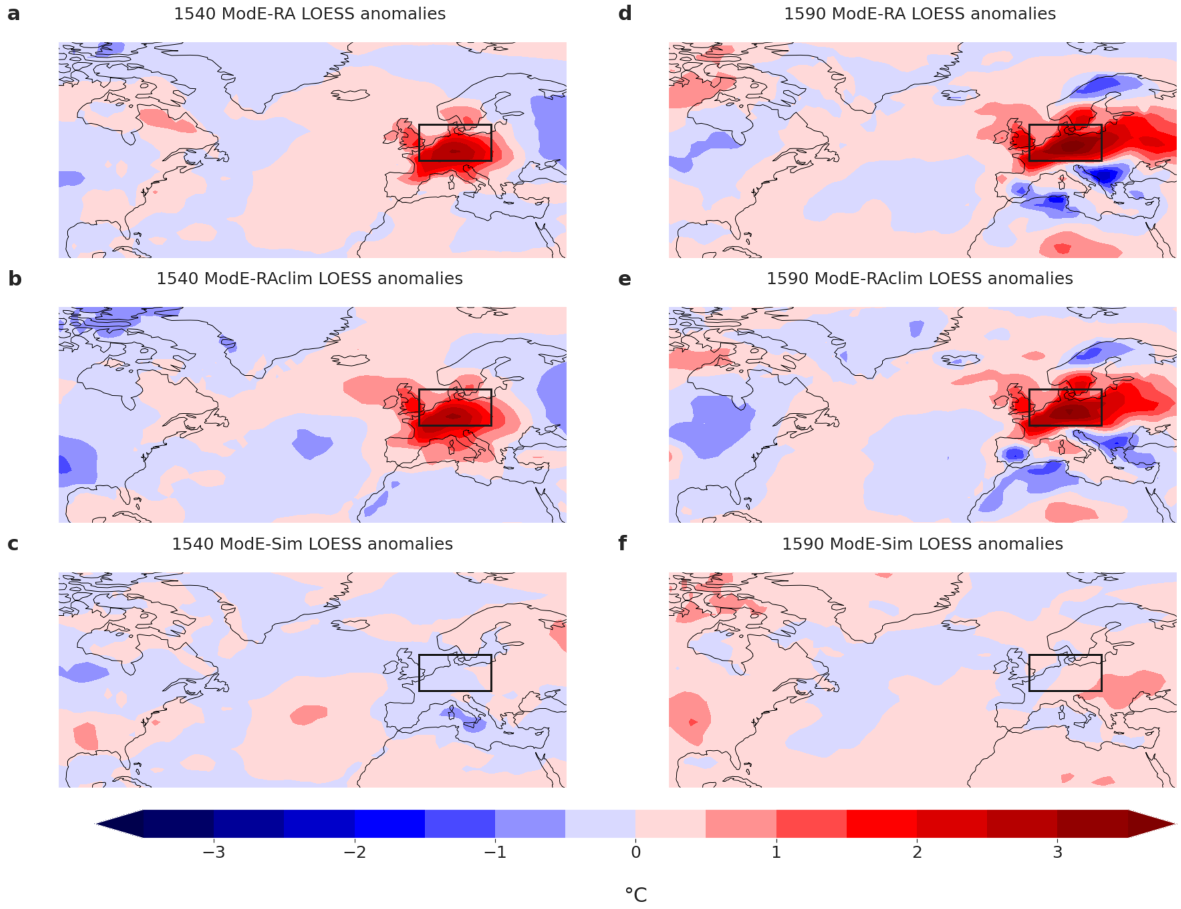

Figure S2: 1540 2m temperature anomalies with LOESS regression for a) ModE-RA, b) ModE-RAclim c) ModE-Sim and 1590 2m temperature anomalies with LOESS regression for d) ModE-RA, e) ModE-RAclim f) ModE-Sim

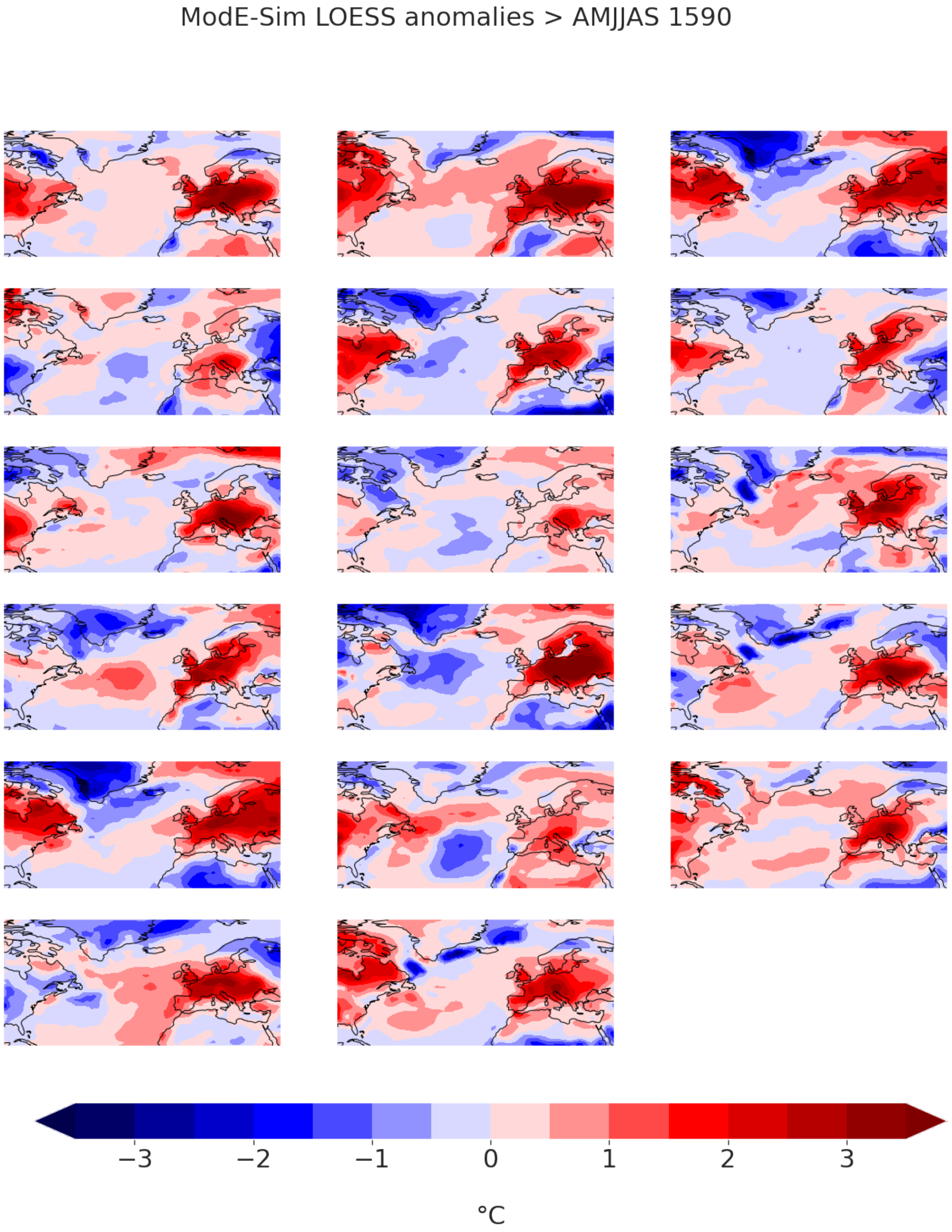

Figure S3: April-September 2m temperature LOESS anomalies for all ModE-Sim years (20 ensemble members) with anomalies higher than 2.17K (1540 AMJJAS anomaly)

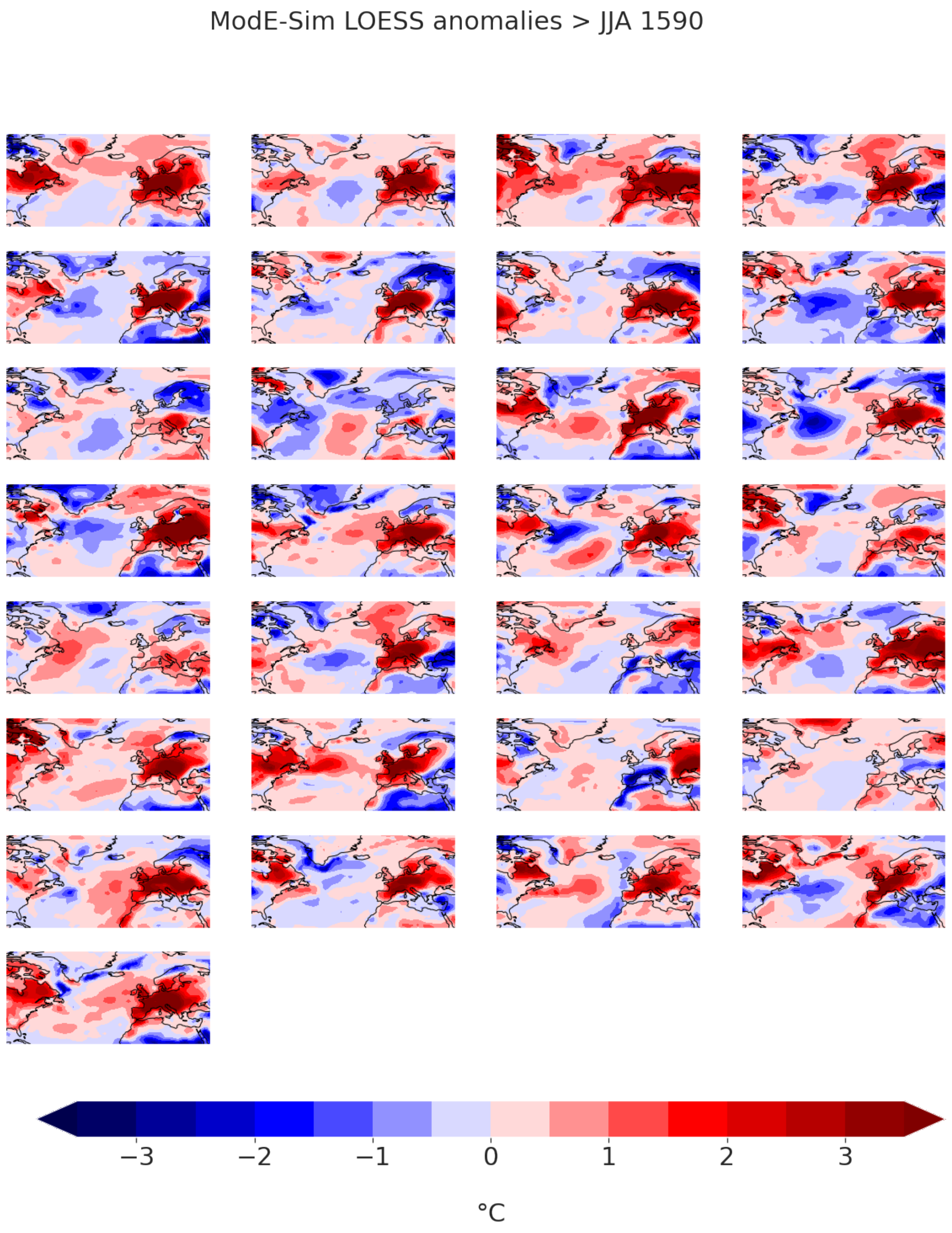

Figure S4: June-August 2m temperature LOESS anomalies for all ModE-Sim years (20 ensemble members) with anomalies higher than 2.8K (1590 AMJJAS anomaly)

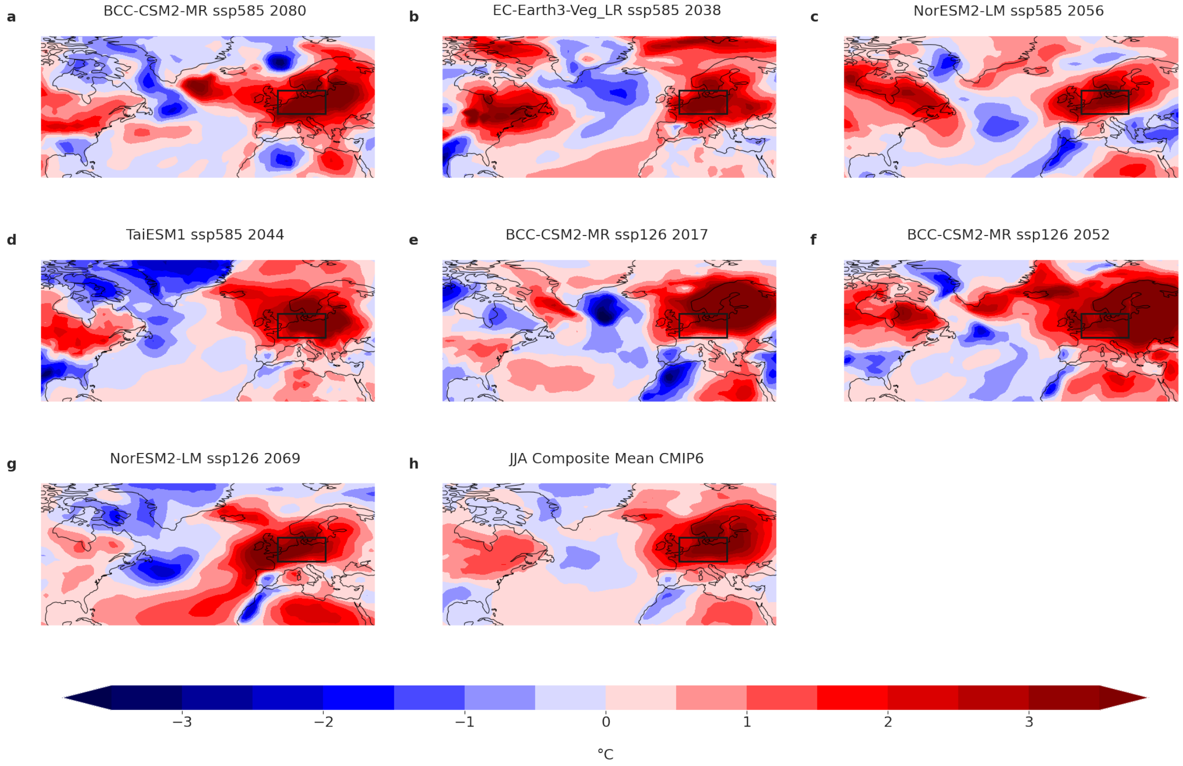

Figure S5: a-g) 2m temperature fields for all CMIP6 simulations with anomalies over Central Europe(0-20°E,47-57°N) greater than ModE-RAs June-August 1590 anomaly (2.8K). h) Composite mean of all CMIP6 simulations with anomalies over Central Europe greater than ModE-RAs 1590 anomaly.

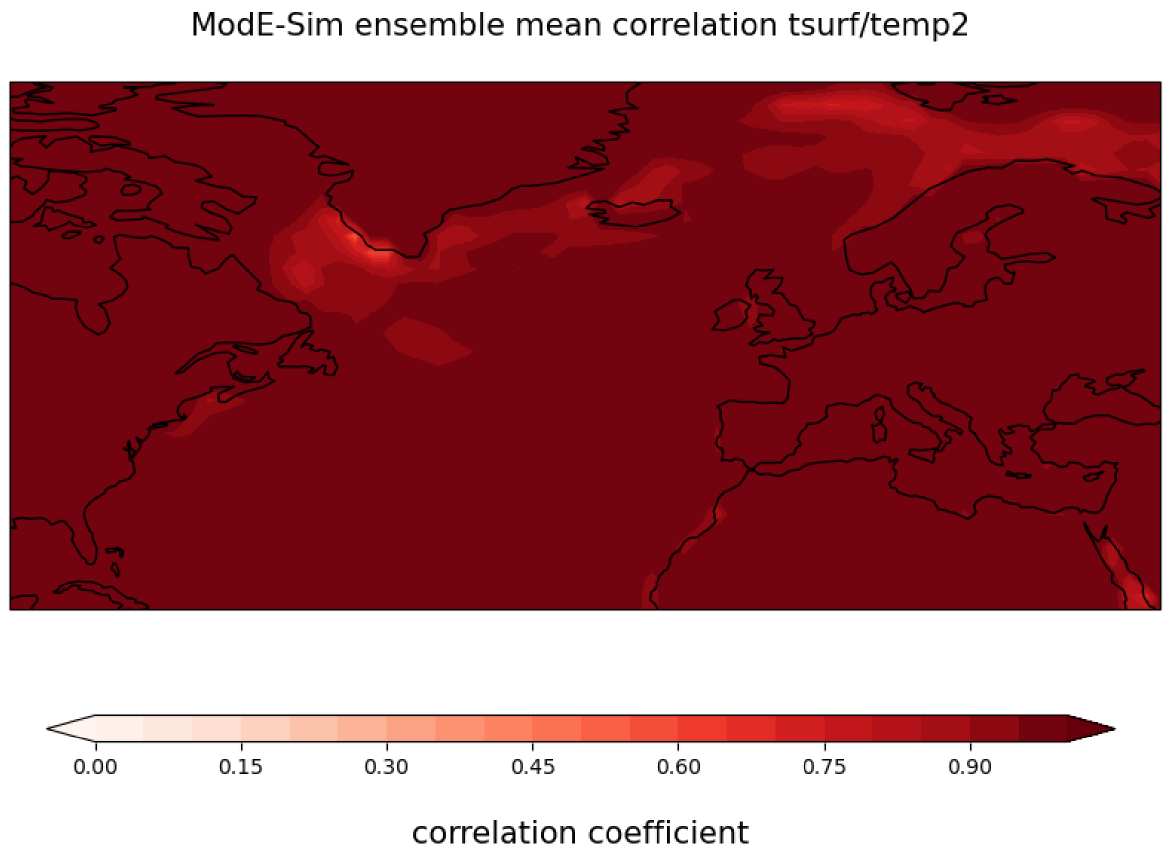

Figure S6: Correlation between surface temperature and 2m air temperature for June-August ModE-Sim ensemble mean
